# A tribute to Dr. Akira Yoshii, MD, PhD

**DOI:** 10.3389/fnsyn.2024.1481435

**Published:** 2024-11-07

**Authors:** Kevin Patrick Koster

**Affiliations:** Department of Neurobiology, The University of Chicago, Chicago, IL, United States

**Keywords:** tribute, to, Dr., Akira, Yoshii

Many members of the neuroscience community have come together to provide a tribute to Dr. Akira Yoshii, M. D., Ph. D., a highly respected clinician and researcher who provided key insights into developmental molecular neurobiology, synaptic protein palmitoylation, and beyond, who sadly passed away in March of 2023. Adding to his extensive direct contributions to our understanding of protein palmitoylation, Akira was central to the compilation of series of quality articles on this topic, which constituted the first volume in this special issue series (https://www.frontiersin.org/research-topics/7997/role-of-protein-palmitoylation-in-synaptic-plasticity-and-neuronal-differentiation/overview). Below, there are memorial messages from his colleagues in neuroscience from different stages of his career in alphabetical order. For a full obituary as well as a more comprehensive list of tribute messages, please see (https://www.tributebook.com/domains/5e93c890-c050-48d6-9c83-682027e01f2d/obituaries/27620075/book).

I first met Akira when he applied for a position as assistant professor in our department. One look at his credentials and he was immediately a finalist. Even then he was a unique combination of basic scientist and clinician. Both his clinical training and his PhD research were at top institutions (MGH and MIT), so I wasted no time in enlisting the Pediatrics Department to make a joint offer and bring him to UIC. There was never a moment of regret, because Akira was not only a brilliant neuroscientist and an outstanding clinician, but he was a warm and generous person. One could not ask for a better choice. As a bonus, he was an exceptional teacher of medical students in Neuroanatomy as well as a mentor for undergraduate and graduate students in his laboratory. He was loved by his colleagues, students and staff. When he was first diagnosed with cancer, almost everyone in the department learned how to make the hundreds of origami cranes that would symbolize our wishes for Akira's recovery. Perhaps they helped a little if only as a reminder that we were all pulling for him. The cascade of cranes was still hanging in his office when his fight against cancer was finally lost. Despite the challenges, Akira continued his teaching, research and clinical practice to the very end. In the last weeks before he passed, he achieved two milestones for an academic scientist: he received a fundable score for what would have been his first R01 grant and he was promoted to Associate Professor with tenure. These achievements were hard won and richly deserved. We only wish he had more time to enjoy the rewards of his hard work. It was not to be. All I can say is that we will continue to miss his brilliance, his gentle manner, his sly humor and his generosity of spirit.

– ***Scott T. Brady, Ph. D., Distinguished Professor, University of Illinois at*** ***Chicago***

We met Akira 15 years ago at MIT. We were postdocs in different laboratories at the Picower Institute. We had the privilege of knowing him and sharing many talks with him in the corridors of building 46, during lunch times, etc. We realized from the 1st day we met that he was a great guy. He was a calm person, very polite, very smart. We had interesting discussions about science, technology, about Japan, Japanese food and traditions, about life. It was always a pleasure talking to him. He was a serious guy but always had a comforting smile when you met him. We shared our passion for neuroscience and our search for becoming independent scientists to pursue our dream experiments. We are totally shocked to receive these sad news. We can't believe he is not going to be around anymore. We send our deepest condolences to his family and friends.

– ***Miquel Bosch, Ph. D., Assistant Professor, International University of*** ***Barcelona and Vicky Puig, Ph. D., Associate Professor, National Research*** ***Council of Spain***

Akira was a wonderful person—kind, considerate, thoughtful, and always willing to help others. He was smart, and wise, and an incredibly good scientist. I was lucky to know him, and to count him as a member of our “lab family.”

– ***Martha Constantine-Paton, Ph. D., Professor Emerita, MIT***

I know of no one who approaches all aspects of life with more sincerity than Akira. As a clinician, as a scientist, as a friend, conversations with Akira have always brought irreplaceable joy and pleasure. Regrettably, I took for granted that these moments would last indefinitely. That was my mistake. I aspire to live out the remainder of my days with the same earnestness that Akira has shown.

– ***Naotaka Fujii, M. D., Ph. D., Hacosco, Digital Hollywood University***

When I look back my life in the U.S. over the past 20 years, I was reminded of how valuable and supportive Akira was in my life. From the fun BBQ parties that he hosted at the roof top of his apartment to the nights where we were both stuck in the labs during the postdoc days at MIT, we spent countless hours together talking, working and pursuing the same professional goal. Nothing changed even after we each started our new path at UMass Medical and UIC. Akira was a true fighter tackling the elucidation of pathophysiological hallmarks of neurodevelopmental disorders both from basic and clinical research aspects. His deep and extensive knowledge in basic and clinical research always reminded me of Akira as an outstanding physician scientist and provided extensive inspiration for my research projects. I learned so much from you, Akira.

His spirit of never giving up will remain in my heart.

I really miss you, Akira!

– ***Kensuke Futai, Ph. D., Associate Professor, UMass Chan Medical School***

You have made a tremendous contribution to our understanding of the synapse. You will forever be remembered in the field of neuroscience. I still remember our conversation about the effect of BDNF on PSD-95 palmitoylation when we were both at MIT almost 20 years ago. I always enjoyed talking with you about our common interest in the synapse and its molecules. I was often inspired by it.

I miss you so much. RIP.

– ***Yasunori Hayashi, M. D., Ph. D., Professor, Kyoto University***

I had the pleasure of getting to know Akira when he joined the MIT lab of my wife Martha Constantine-Paton in 2000. I had the added pleasure of collaborating with and co-authoring a research paper with Akira a few years after that. Akira was special. As I wrote in a letter of recommendation for him, Akira's “intellect, encyclopedic knowledge, dedication to his patients and to science, and ability to excel in the clinic, in teaching, and in the research laboratory” placed him in the top tier of physician-scientists. But more fundamentally, Akira was a kind, wonderful, and generous human being, always thinking about and placing the interests of others above his own. I was happy that we stayed in contact after Akira moved to Chicago. I will miss him very much.

– ***H. Robert Horvitz, Ph. D., David H. Koch Professor, MIT***

When I was at MIT, I heard about a wonderful Japanese guy named Akira-san, who works as both a researcher and a medical doctor. He was a bit shy, but generously shared his advice about life and research, especially at BBQ at his apartment.

The year before he passed away, I had a chance to talk with him. He asked me in his usual tone of voice, “What are you doing now?” I explained my ongoing research, and we had a fruitful discussion. He mentioned his disease but was more concerned about his research and NIH grant. He said, “Please don't tell anyone else about my disease,” not wanting to worry others. He was truly strong and kind.

I never imagined that he would be gone so quickly. I miss Akira-san so much.

May you rest in peace, Akira-san.

– ***Takao Keizo, Ph. D., Professor, Toyama University***

I would wager with confidence that Akira was among the absolute top neuroscientists in terms of his dedication to understanding the brain's inner workings. My confidence comes from the way Akira lived his life—in monk-like dedication to doing and applying science. His home was modest and simple. His life was also deliberately simple. It is as if he could have no distraction from his goals, which were both purely curious and humanitarian. From the time I met him until his body began to fail to an extent that he could not leave his house, he kept the same routine. He arrived at work in the morning, checked to make sure the lab was busy, proceeded to work until 5:45 pm, when he would check on the lab again, and then continue to work for another few hours. Akira worked 7 days a week, 365 days a year, for the entire time I knew him and, as I learned from others, his entire adult life. The mastery over his will is a testament to the character Akira shed onto everyone he met. It is virtually impossible to make a greater effort than he did.

To know Akira was to know how profoundly dedicated he was to science. He seemed to know everything about everything. And not just on a surface level but deeply, and from multiple perspectives. This is in part why he was so revered both as a research scientist and as a clinician. I have not met another soul who took the scientific endeavor as seriously as Akira did—the example he set as a scientist, scholar, highly creative researcher, critical thinker, and research team leader is truly remarkable. It is impossible to replicate the level of Akira's accomplishments while maintaining his robustness of character and yet, he always did his best to bestow us, his trainees, with the necessary qualities to maximize our potential. Perhaps just as importantly, Akira had great tact as a mentor. He knew when and how to push for more but also when to be the gentle, compassionate person that was ever present under the surface of his more stoic disposition.

Akira and I shared more than 5 years working together and I will forever be grateful for my time with him. He taught me more than I can hope to learn through the rest of my career. I will dedicate every success in my career in some way to Akira's mentorship and my foremost goal is to honor his legacy with top-notch scientific discoveries. I can only hope to be half of the person Akira was, but I will try my best to honor the hardest working person I have ever met.

– ***Kevin P. Koster, Ph. D., Postdoctoral Scholar, University of Chicago***

In the Department of Anatomy and Cell Biology, we all carry the memory and amazing legacy from Akira deep in our hearts. His passion for science was simply amazing, and the way he dedicated his life to advance is exemplary. On a more personal level, watching him endure pain so stoically deeply impacted my own perspective of life forever. We will also miss the great times we shared while enjoying sushi or ramen, his favorite meals. Rest in Peace, dear Akira.

– ***Gerardo Morfini, Ph. D., Associate Professor, University of Illinois at*** ***Chicago***

We got to know each other probably in 2000, just before you joined the lab of Martha Constantine-Paton at MIT. Since then, you took me to lunch café and yummy restaurants near the school. After the daily experiments done, we often went to the drink bars and discussed the functions of synapses, PSD-95, and glutamate receptors. As if you got married with, you devoted yourself to research and worked so hard. Your dream came true in a few years. You got a faculty position at University of Illinois Chicago and quickly built up your own lab. And you discovered an interesting novel mechanism in synaptic physiology that made you an NIH R01 grantee. I think that during your last few years, fighting against cancer, you showed us how man's life is dedicated to his/her dream.

I will miss you very much, Akira!

– ***Kazu Nakazawa, M. D., Ph. D., Asaka Hospital***

Since I moved to the University of Illinois at Chicago as a young faculty member in 2018, Akira has become one of my closest friends, the best collaborators, and the scientists who encouraged me by showing his fighting spirit whenever. It was my treasure. Without his contributions, I would have missed many significant findings in our collaborative studies. He also helped and saved me even when he had challenging times. He is absolutely phenomenal, and I am delighted to meet and work with Akira. His contributions stay forever in our minds, and his achievements continuously provide us with tremendous benefits for ongoing science.

– ***Teruyuki Sano, Ph. D., Assistant Professor, University of Illinois at Chicago***

Akira was one of the most engaging and inspiring young scientists that I met at MIT. His passion, hard work, and kind spirit left strong impressions on me. I really enjoyed our collaboration on PSD-95 translocation during eye opening in visual system development, and I feel proud to be on that paper (Akira Yoshii et al PNAS 2003).

Martha Constantine-Paton always said wonderful things about Akira, and I have to agree. Akira was a rare scientist who was rigorous, thoughtful and creative—and a lovely person on top of that!

I will miss Akira-san.

Sincere condolences to the family.

– ***Morgan Sheng, M. D., Ph. D., Professor, Broad Institute, MIT***

I had the privilege to know Akira for 6 years after I joined the Department of Anatomy and Cell Biology at UIC in 2017. Because of his research interests in synaptic plasticity and brain development, we started organizing regular meetings with his lab members to discuss new ideas and collaborative projects. Akira's dedication to science, collegial attitude, and dedication to students was exemplary and will forever be remembered.

– ***Kuei Tseng, M. D., Ph. D., Professor, University of Illinois at Chicago***

Perhaps the most poignant summary of Akira's contribution to neuroscience, his patients, and his collaborators comes from his clinical colleague, Dr. Leslie Jabine, who said, simply—“no one can do more.”

We will all miss you, Akira.

